# An Unusual Hunting Accident: A Case Report

**DOI:** 10.7759/cureus.66911

**Published:** 2024-08-15

**Authors:** Beáta Á Borsay, Barbara D Halasi, Róbert K Pórszász, Katalin Károlyi, Péter A Gergely

**Affiliations:** 1 Institute of Forensic Medicine, Faculty of Medicine, University of Debrecen, Debrecen, HUN; 2 Department of Pharmacology and Pharmacotherapy, Faculty of Medicine, University of Debrecen, Debrecen, HUN; 3 Department of Pathology and Laboratory Medicine, Jefferson Einstein Hospital, Philadelphia, USA

**Keywords:** distant-range shot, long-range shot, unintentional hunting accident, far distant discharge, ballistics, shotgun injury, forensic medicine, medico-legal autopsy, rifle accident

## Abstract

In Hungary, possessing certain weapons (e.g. firearms, bows, air pistols, and air guns mainly over 7.5 Joule muzzle velocity) is strictly regulated. In case of firearm deaths, in our country, we usually have to consider the role of military personnel or a hunter. Getting a game license for five years is a complex procedure. Class participation in weapon training, hunter ethics, and rules, first aid, manners of hunting, wild animals' knowledge, wildlife management, game laws, etc. is compulsory besides a prosperous exam at the Hunt Authority. A psychological license is also mandatory. Through permission from the police for a firearm license, buying weapons for hunting is possible. The storage of firearms and cartridges is rigidly controlled and checked. Some special types of hunting (e.g. with a bow, bird of prey) require additional licenses. The fact and the duration of the hunt and all shoots should be registered. The authors report an extraordinary fatal hunting accident because of non-regulation rifle (Blaser R8 338) use (unintentional shot), in which the travel distance of the projectile was more than 2000 m and the victim suffered fatal injuries at his daughter’s homeyard. This was a non-target, extreme long-range shot. The ethical range in hunting is within 150 m, a practiced hunter with proper precision tools can shoot accurately within 300-400 m and in extremely rare cases within 700-800 m. Military snipers can operate over a 1000 m distance. Even if this was a targeted shot (with a 2161 m range), not many professionals would have been able to aim at the target. A sequence of accidents was necessary for this fatal case. With the application of X-ray examination and a special layer-by-layer method of forensic autopsy, the bullet, the entry wound, the primary shot channel, and lethal injuries have been revealed. With the help of the found projectile and the rigorous hunting regulations, the alleged perpetrator was identified within a short time.

## Introduction

One of the main special fields of interest in forensic or medico-legal autopsies is accidental death due to a non-criminal act. In Hungary, the number of deaths due to the use of some weapon releasing a moving object with different amounts of kinetic energy, either in intentional or unintentional form, is relatively rare, in contrast to certain countries [1,2]. Kinetic energy is influenced by the mass and velocity of the projectile [3]. This energy is absorbed by the target tissues, resulting in a missile wound. A projectile with the same kinetic energy, depending on the affected body parts, can cause relatively minor, life-threatening, or fatal injuries [4]. In the case of shot-related death, a forensic autopsy must be performed.

## Case presentation

In the present case, the victim was a 59-year-old half-pay officer who stood in one of his daughters' yards and was checking the fence. After a thump, he suddenly collapsed, complaining of sharp abdominal pain. He lost consciousness, and within 10 minutes, the non-emergency medical on-call service and paramedics were on the scene. The patient had no measurable blood pressure, Arterenol and isolyte admission was needed besides oxygen application; thus, after stabilizing his condition, he was transported to the Emergency Department. In the hospital, the medical attendance was confined to cardiopulmonary resuscitation, and within a short period, he died. After the farewell of their beloved, the relatives were returned the deceased’s clothes by the hospital. On the way home, his wife hugged his sweater and checked it since during leave-taking, she noticed some blood on the back of his late husband. She realized that there was a round hole on the back of the sweater on the left side. They immediately returned to the hospital to inform doctors who acquainted the police with the suspicion of a lethal gunshot injury.

**Figure 1:**
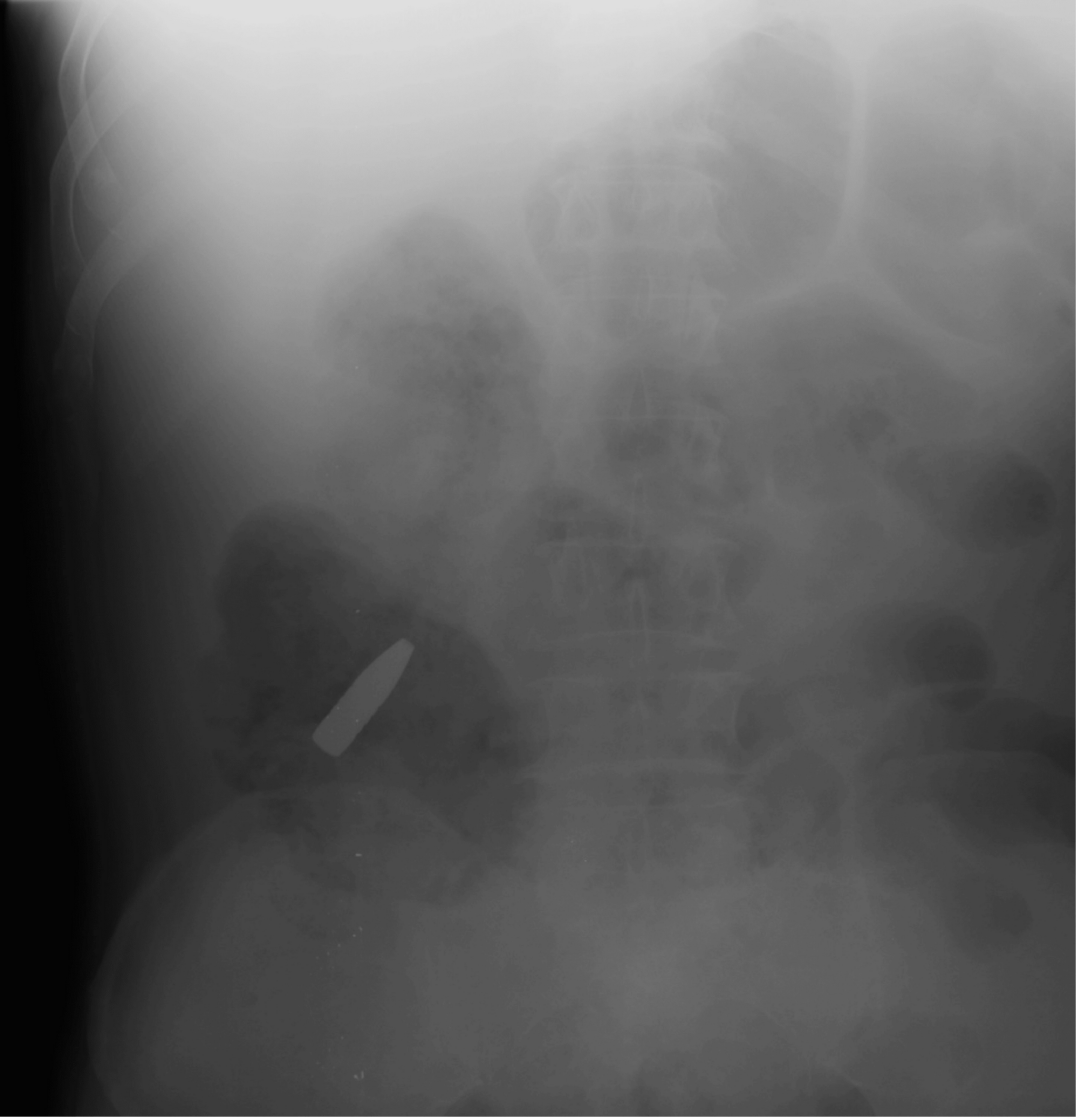
AP (anteroposterior) X-ray examination preceding the autopsy indicating the position of the bullet

**Figure 2:**
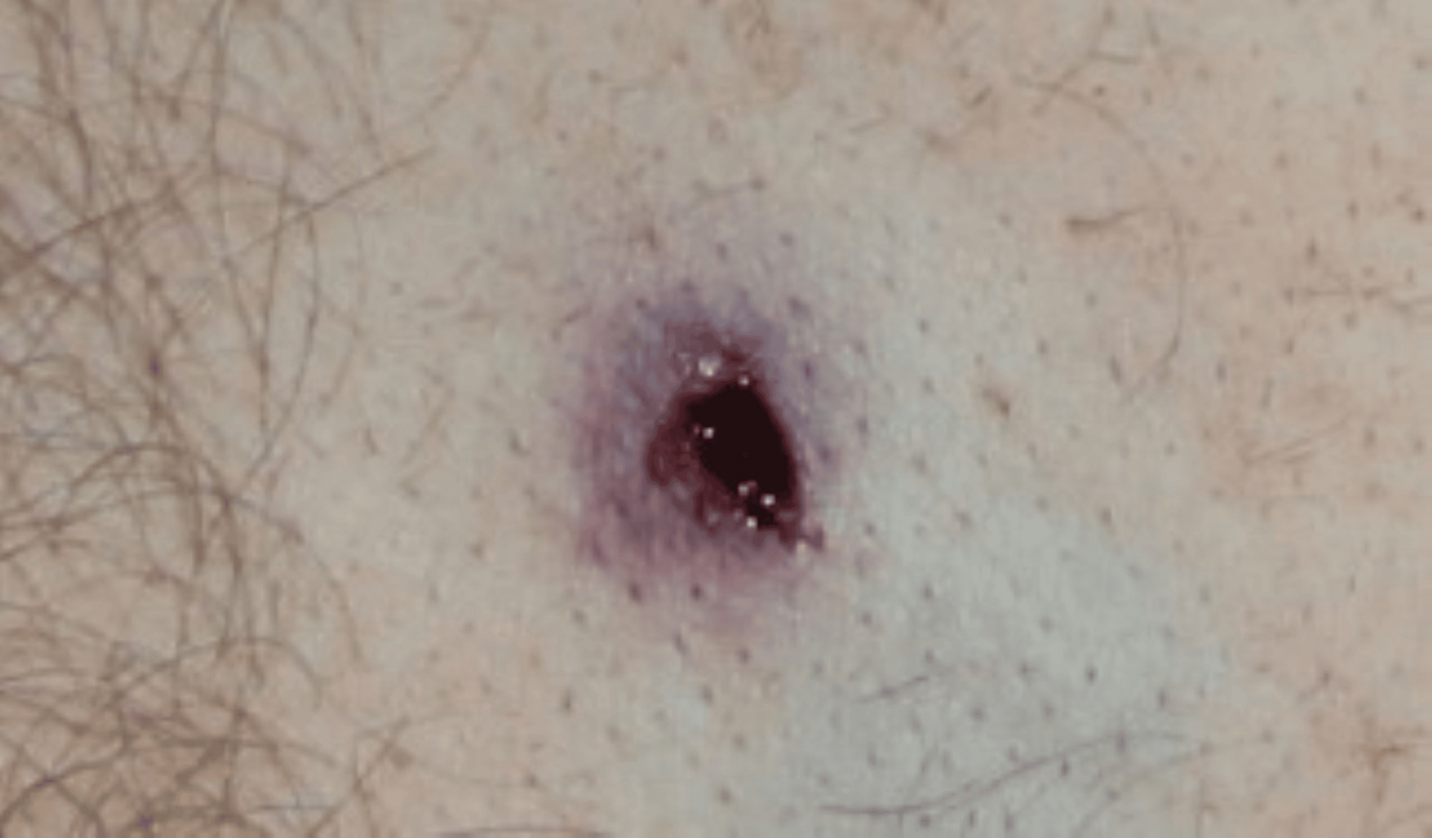
The atypical entry wound on the corpse The irregular shape, partly irregular edges, and contused rim were noticed as a consequence of the unstable ammunition impact. It also indicated that the projectile was in its terminal ballistics stage and referred to a distant-range shot.

**Figure 3:**
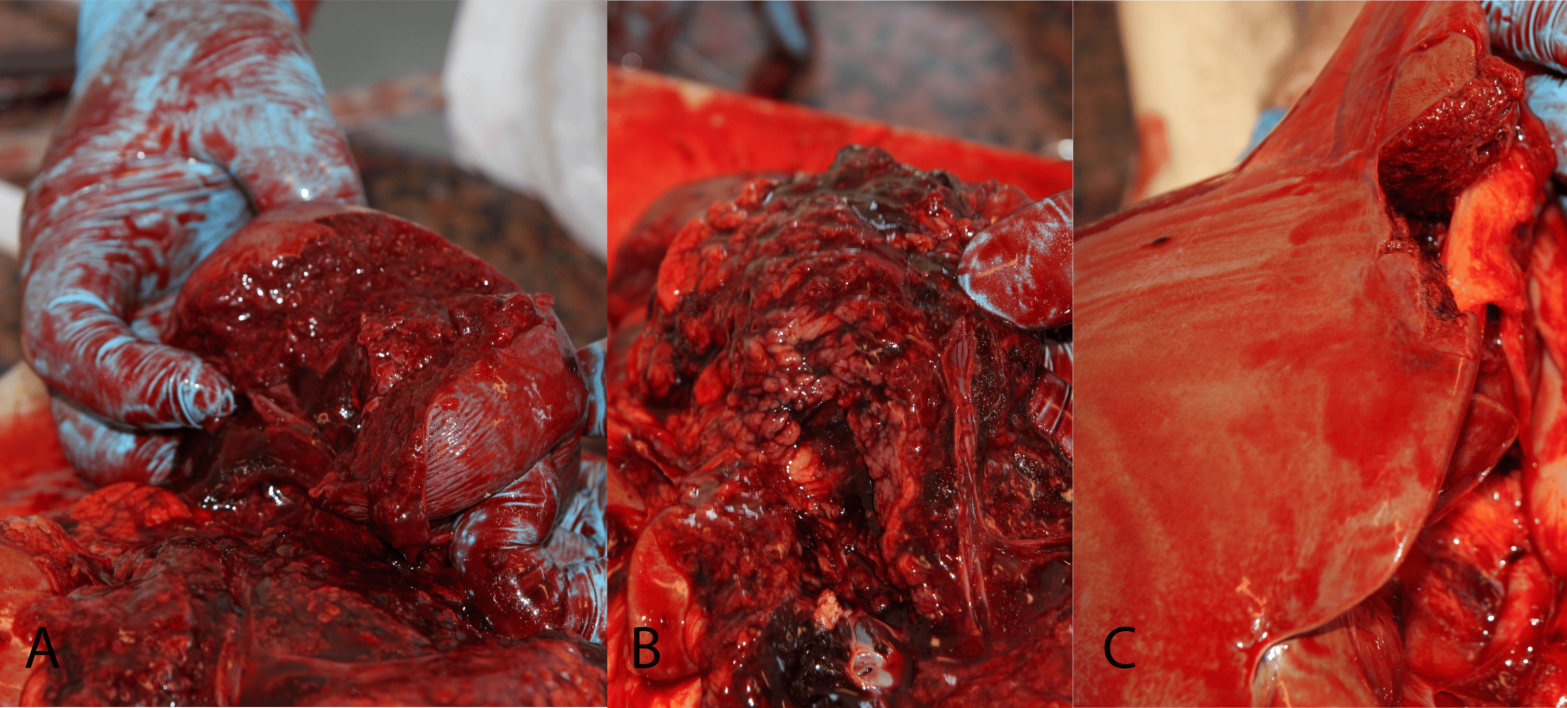
Destruction of the organs alongside the shot channel (A) Explosion of the spleen. (B) Rupture and hemorrhage of the pancreas. (C) Discontinuity of the liver.

**Figure 4:**
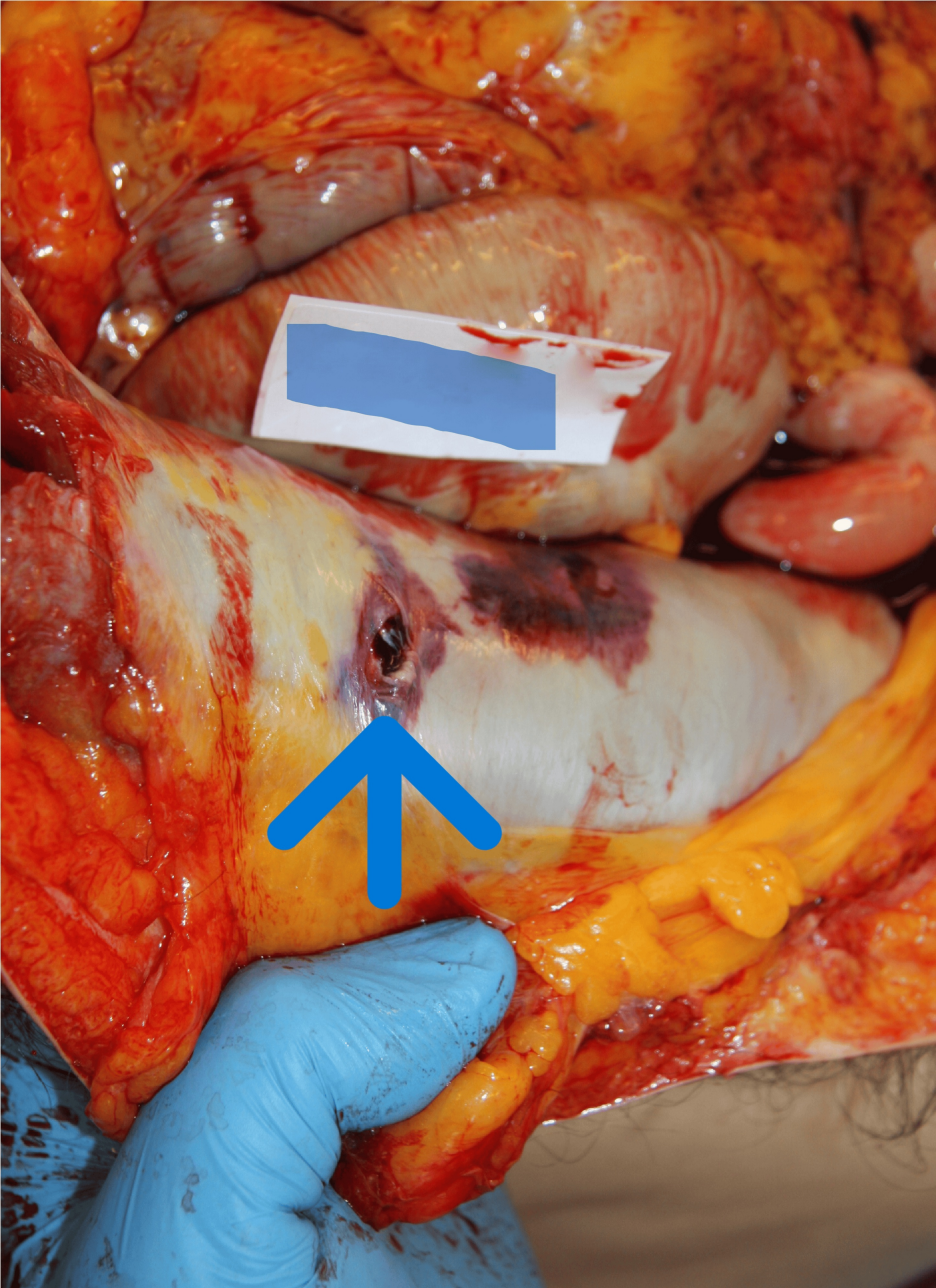
The abdominal wall at the end of the shot channel where the missile was found

**Figure 5:**
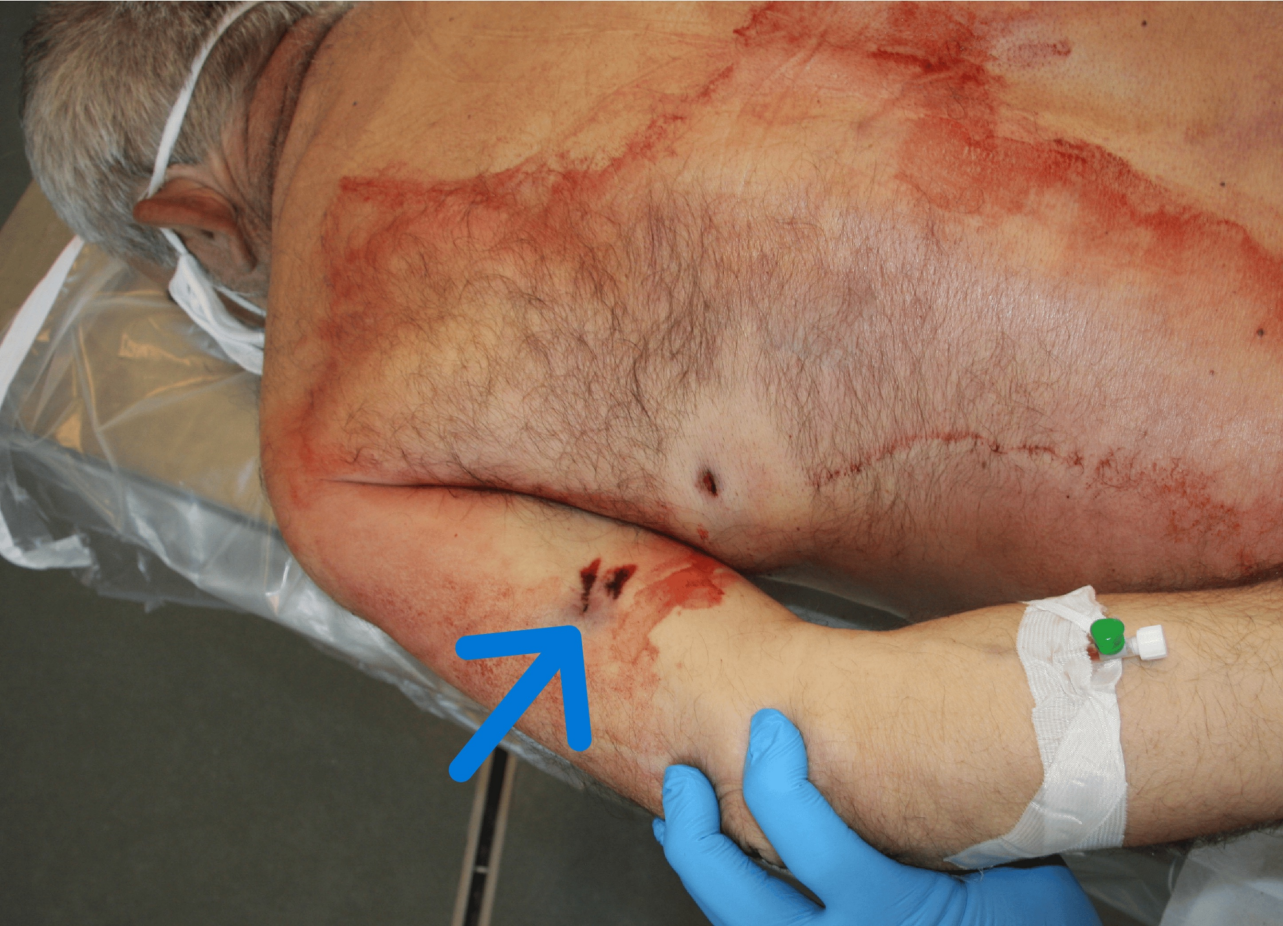
Posterior side of the left upper arm Bruises and contusion were seen as a consequence of a tangential effect of a bullet (arrow) with the entrance wound on the back.

An urgent autopsy was performed. Postmortem CT is not available in Hungary; however, an PC-ray preceding the examination revealed a metallic projectile on the right side of the abdominal cavity (Figure 1). The entry wound was a bit irregularly shaped, with partly irregular edges, 4×6 mm in diameter, and skin tissue loss with a 3-4 mm contused rim from 130 cm of the level of the sole and 19 cm left from the midline in the area of the scapula (Figure 2). No abrasion ring or soot deposition was observed. A layer-by-layer autopsy technique was applied to follow the shot channel. It went into the left thoracic cavity between the 10th and 11th ribs, through the diaphragm, injuring the spleen, via the pancreas tail and, the left adrenal gland, above the lesser curve of the stomach into the perigastric fatty tissue over the left lobe of the liver reaching the peritoneum. After entering the right rectus abdominis muscle (107.5 cm from the level of the sole and 6 cm right from the midline), the bullet turned down and back but did not enter the abdominal cavity again. It made a sharp bend (Figure 3, Figure 4). The bullet was recovered and, without cleaning, was consigned to the police. The direction of the shot channel was from left to right, back to front (posteroanterior), and above to down (craniocaudal). As a consequence of internal organ injuries, 1400 ml blood was observed in the abdominal cavity, and as a concomitant anomaly, parallel, line-shaped bruises and suffusion of the posterior side of the left upper arm below 9.5 cm and 11 cm from the axilla region were noted (Figure 5). Histological examination of the entrance wound showed no evidence of powder-burn particles, in contrast to close-range gunshot injuries [5]. We determined traumatic shock as a cause of death. As for natural diseases, prostate gland enlargement and left ventricular hypertrophy have been observed. Toxicological analysis excluded the presence of alcohol in the postmortem blood and urine samples.

**Figure 6:**
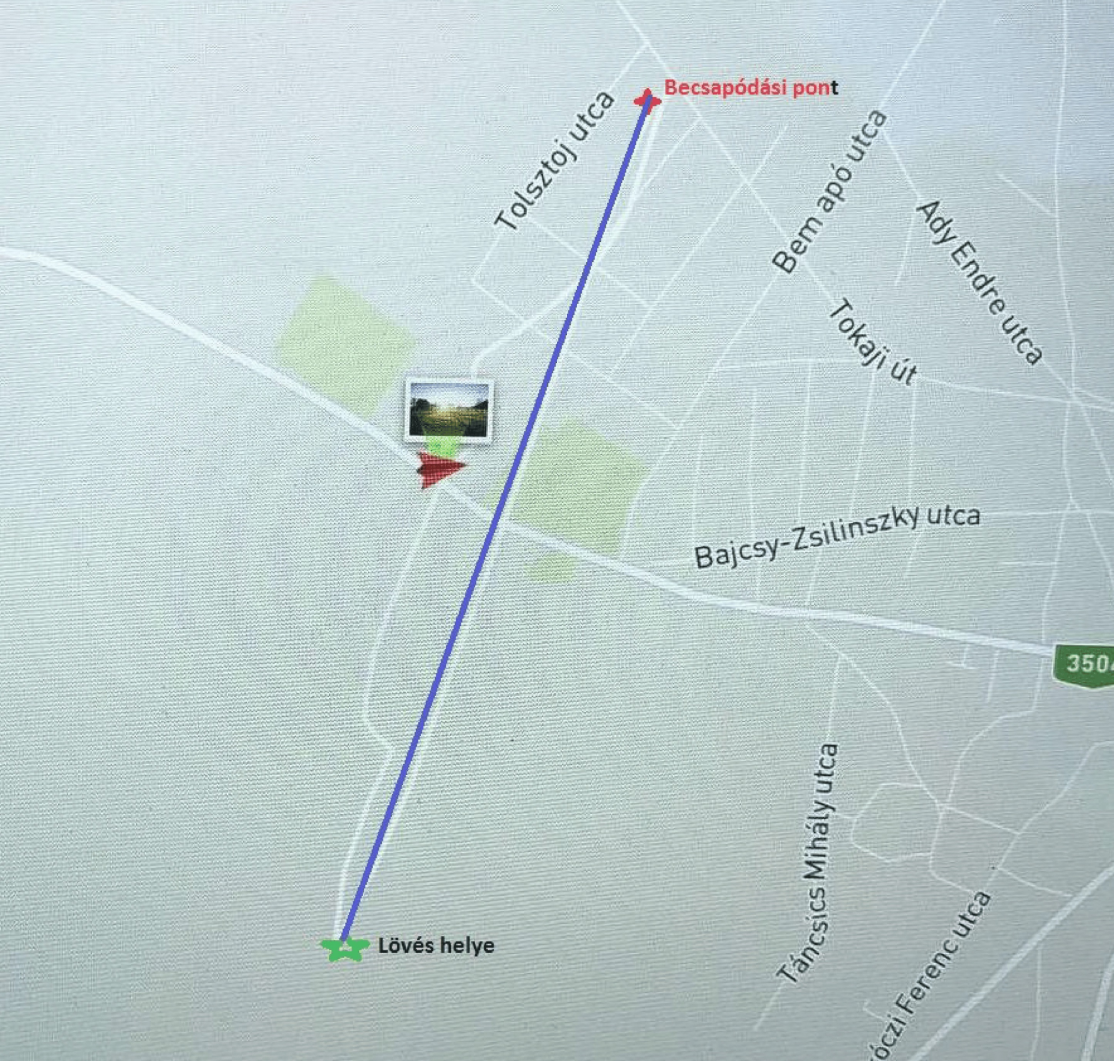
The drone record about the shot range The green star represents the place of the shot, the red cross indicates the impact point of the missile and the blue line demonstrates the 2161-meter distance of the discharge.

**Figure 7:**
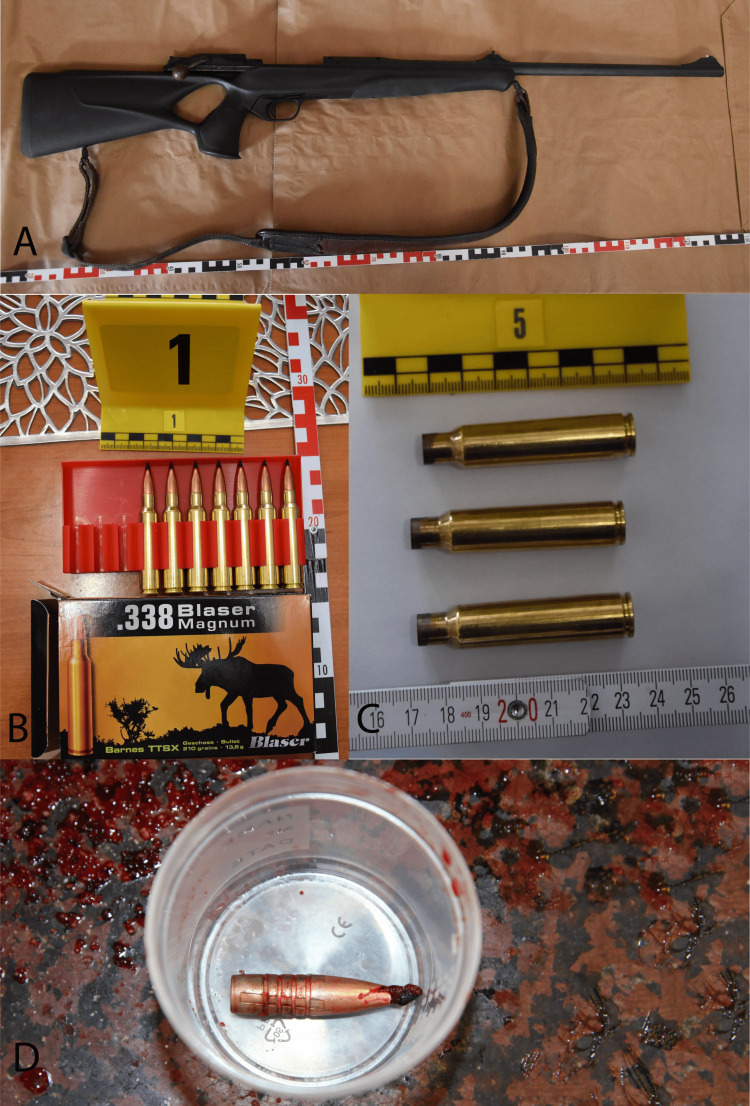
Seized rifle, cartridges, and bullets (A) Seized Blaser R8 338 rifle as a lethal weapon. (B) Original cartridges as booked evidence. (C) Engaged cases. One of them was the case of the fatal ammunition. (D) The forensic pathologist found the bullet during the autopsy.

Not far from the crime scene (935 m), a hunting ground was situated (Figure 6). According to the above-mentioned, all hunting is registered in Hungary, narrowing the range of possible perpetrators, to only one person left. Perquisition was held by the police, and the potential weapon, cartridge, and cases were booked (Figure 7). A forensic ballistics expert and a forensic hunting expert were involved in this case. The reserved weapon was a Blaser R8 338 with a known gun barrel-, holder,- and lock serial numbers. The cartridges were " .338 Blaser Magnum '' caliber signed, " norma .338 Blaser Mag '' bottom signed, with a lead core, copper jacketed, plastic tipped, Barnes-made, TTSX type with 13.57 g mass each. The muzzle velocity (V0) was 900 m/s and the muzzle energy was 5514 Joule. The bullet was identified and examined by a forensic ballistics expert. It exhibited special characteristic features (so-called ballistic fingerprints) which allowed the identification of the weapon via probe shot and microscopical examination that matched with the aforementioned.

**Figure 8:**
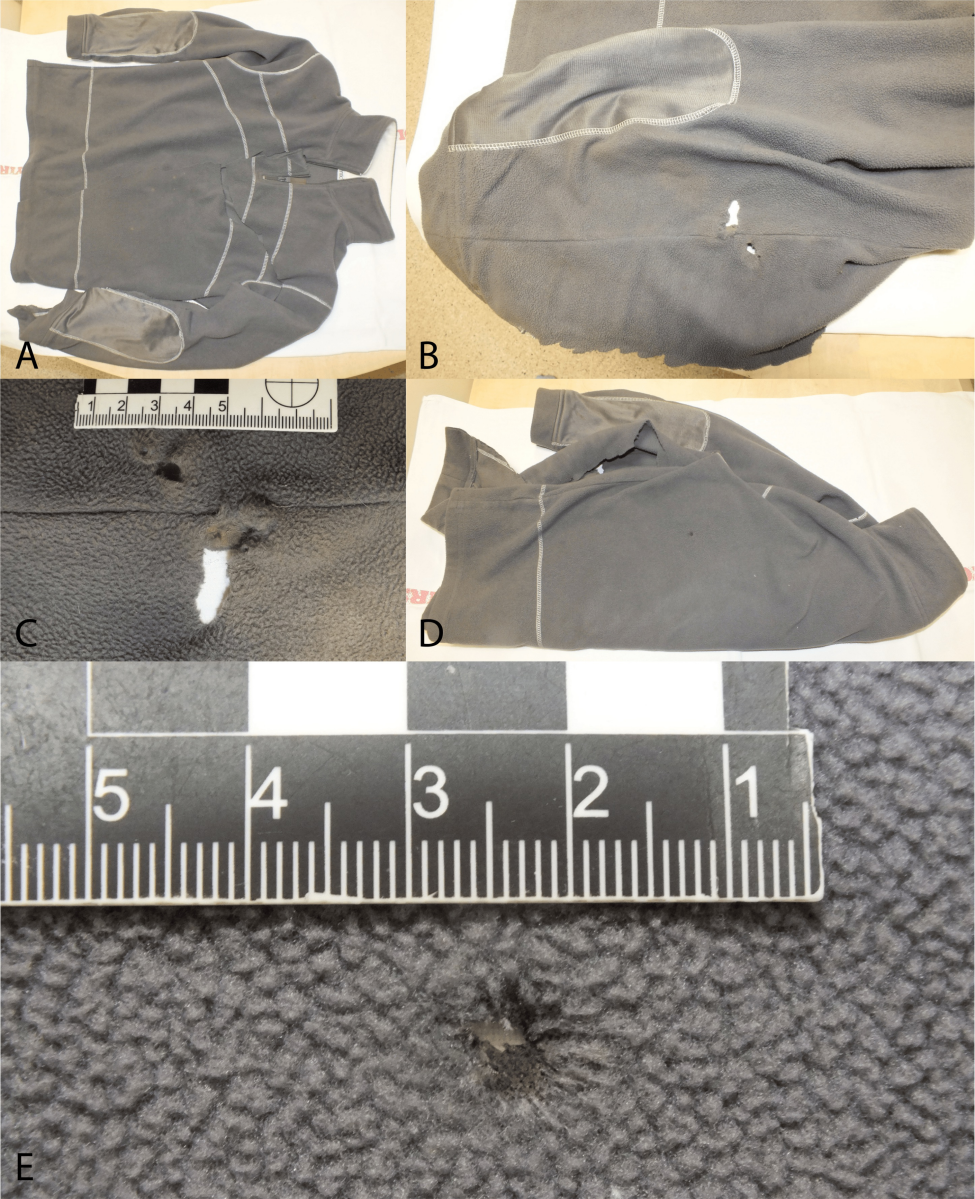
The sweater of the victim was removed (cut off from afresh) by the paramedics during the resuscitation (A) An overview picture of the sweater of the deceased from the anterior view with the artificial cut. (B) The posterior side of the left upper arm was injured and the cloth also sustained damage. The mechanical effect of the ammunition, because of the polyester content of the material and the proper kinetic energy, was accompanied by the heat effect. (C) Closer look at the left sleeve. (D) The left-back side of the pullover where a missing material was detected in the projection of the entry wound of the corpse. (E) A close-up of the previous image. A round-shaped missing material was observed with a blood-polluted area.

Test firing is a beneficial method generally, and it was in this case [6]. The rifle operated flawlessly; according to the investigational data, the fatal shot was a result of bungling and not of an intentional act. Considering the environmental factors (e.g., relative humidity, temperature, direction, and velocity of the airflow), the angle of the shot, and the fact that the decelerating missile lost its stability, the exact trajectory was erratic. The missile lashed to the body from up to down presumably under a 30-40° angle compared to the horizontal direction (according to the opinion of the forensic ballistics expert). As the actual trajectory could not have been calculated, the determination of the kinetic energy upon impact was also undefinable. It was a far shot or far distant discharge; therefore, the impression of the muzzle or smoke soiling was not detectable around the entrance wound. The range was 2161 m; thus, the hunt itself was also ruleless because the safety distance in the case of using a rifle from a built-up area is at least 5-6000 m according to the “Law No. LV. of 1996 on the protection, management, and hunting of wildlife”. The perpetrator was a guest tracker for the firearm owner. Examination of the cloth also yielded useful information regarding the injury of the left sleeve and posterior side (Figure 8).

## Discussion

Using the layer-by-layer technique during the autopsy, the cause of death, the primary shot channel, and the projectile were determined. In most of these cases, cooperation between the different types of forensic scientists and the police is essential to clarify the manner of death in cases of unnatural death, so it is a combined teamwork. Extensive inspection of corpses and clothes is mandatory in forensic autopsies. The application of post-mortem imagery is also useful and compulsory (if it is available) in gunshot death, in addition to forensic ballistics and other necessary forensic experts, for a better understanding of macroscopic autopsy findings. The range of procedural options of forensic pathologists is limited without a postmortem CT scan method or X-ray examination, so the improvement of the "virtopsy” techniques would be desirable not only in the case of gunshot wounds but also routinely.

Two basic rules must be kept to avoid such lethal accidents: always keep the firearm off-load until ready to use and always keep the weapon pointed in a proper direction or under angle. Similar celebratory gunfire injuries were reported from the United States (U.S.) when fired into the air during these occasions and the "falling bullets” caused severe injuries or fatalities [7]. This case report presents a less common and unusual case of firearm death. According to statistical data from 2010 to 2022 in Hungary, the unintentional form comprises 1.75 % of all gunshot deaths [8]. In Hungary, firearm laws are relatively strong. The number of unregistered weapons is comparatively high, and the evaluated firearm ownership is approximately 10.5/100 inhabitants [9]. The number of firearms with gun licenses in Hungary is approximately 222,000 (⁓2.3/100 people), so the others are non-license requiring firearms or illegal firearms. Nobody knows the total number of weapons and illegal firearms that pop up occasionally within Hungary or in other countries in the region. Unintentional gunshot deaths typically bond to registered weapons and hunting acts, as in our case [10,11]. This kind of long-distance, nontargeted, fatal, unintentional firearm death case has not been observed so far in our institute, and other types (low trajectory, impolitic) are typical.

## Conclusions

In the case of sudden death, the possibility of not being a usual mode of death must be considered. Knowledge of the circumstances is cardinal in forensic medicine to determine the manner of death. This unique, unintentional, hunting-related firearm death case report highlights the importance of cooperation between different types of forensic experts to correctly classify the case. Although firearm control regulation is stringent in Hungary, in this case, it could not prevent a fatal accident but was able to support law enforcement by narrowing the range of the culprit. The suspected perpetrator was found within 24 hours of the incident. Adherence to hunting regulations would have been sufficient to prevent such accidents. The proper and rigid law framework cannot guarantee rational gun usage and individual liability is also part of the formula. The significance of firearm safety education cannot be emphasized sufficiently, which is the responsibility of all firearm owners, firearm manufacturers, authorities, and firearm safety instructors.
